# Systematic transcriptome analysis of the zebrafish model of diamond-blackfan anemia induced by RPS24 deficiency

**DOI:** 10.1186/1471-2164-15-759

**Published:** 2014-09-04

**Authors:** Binfeng Song, Qian Zhang, Zhaojun Zhang, Yang Wan, Qiong Jia, Xiaomin Wang, Xiaofan Zhu, Anskar Yu-Hung Leung, Tao Cheng, Xiangdong Fang, Weiping Yuan, Haibo Jia

**Affiliations:** Key Laboratory of Molecular Biophysics of Ministry of Education, College of Life Science and Technology, Center for Human Genome Research, Huazhong University of Science and Technology, Wuhan, Hubei 430074 China; CAS Key Laboratory of Genome Sciences and Information, Beijing Institute of Genomics, Chinese Academy of Sciences, Beijing, 100101 China; State Key Laboratory of Experimental Hematology, Institute of Hematology and Blood Diseases Hospital, Chinese Academy of Medical Sciences & Peking Union Medical College, Tianjin, 300020 China; Department of Medicine, University of Hong Kong, QMH 418 Hong Kong, China

**Keywords:** DBA, hematopoiesis, miRNA-seq, RNA-seq, RPS24

## Abstract

**Background:**

Diamond–Blackfan anemia (DBA) is a class of human diseases linked to defective ribosome biogenesis that results in clinical phenotypes. Genetic mutations in ribosome protein (RP) genes lead to DBA phenotypes, including hematopoietic defects and physical deformities. However, little is known about the global regulatory network as well as key miRNAs and gene pathways in the zebrafish model of DBA.

**Results:**

In this study, we establish the DBA model in zebrafish using an *RPS24* morpholino and found that *RPS24* is required for both primitive hematopoiesis and definitive hematopoiesis processes that are partially mediated by the p53 pathway. Several deregulated genes and miRNAs were found to be related to hematopoiesis, vascular development and apoptosis in *RPS24*-deficient zebrafish via RNA-seq and miRNA-seq data analysis, and a comprehensive regulatory network was first constructed to identify the mechanisms of key miRNAs and gene pathways in the model. Interestingly, we found that the central node genes in the network were almost all targeted by significantly deregulated miRNAs. Furthermore, the enforced expression of miR-142-3p, a uniquely expressed miRNA, causes a significant decrease in primitive erythrocyte progenitor cells and HSCs.

**Conclusions:**

The present analyses demonstrate that the comprehensive regulatory network we constructed is useful for the functional prediction of new and important miRNAs in DBA and will provide insights into the pathogenesis of mutant *rps24*-mediated human DBA disease.

**Electronic supplementary material:**

The online version of this article (doi:10.1186/1471-2164-15-759) contains supplementary material, which is available to authorized users.

## Background

Diamond Blackfan Anemia (DBA, OMIM105650), which presents in infancy, is a rare congenital hypoplastic anemia characterized by the marked heterogeneity of clinical manifestations, such as anemia, macrocytosis, reticulocytopenia, developmental abnormalities, growth retardation and an increased risk of malignancy [1, 2]. Mutations in ribosomal protein genes ((encoding proteins for 40S ribosome (RPS) or 60S ribosome (RPL)) in humans have been identified as the causes of DBA because ribosome biogenesis is indispensable for immature erythrocytes in early and rapid growth phases [3]. For example, the human RPS19 gene, which encodes a protein that is part of the small subunit of the ribosome, was the first to be identified and the most frequently mutated ribosomal protein gene, accounting for approximately 25% of DBA patients [4]. Mutations in *RPS24* and *RPS17* have been found in approximately 2% of patients [5, 6], while mutations in genes encoding large ribosomal subunit-associated proteins, *RPL5*, *RPL11* and *RPL35A*, have been reported in 9% to 21.4%, 6.5% to 7.1%, and 3.3% of patients, respectively [7, 8]. To date, approximately 50% of DBA patients have been found to have at least a single heterozygous mutation in a gene encoding a ribosomal protein [9, 10].

*RPS19*, *RPL11* and *RPS24* insufficiency cause distinct cell cycle defects in DBA patients [11, 12]. RPS19 mutations decrease the proliferation of progenitor cells; however, terminal erythrocyte differentiation remains normal, with little sign of apoptosis. In contrast, *RPL11* mutations lead to a dramatic decrease in progenitor cell proliferation, delayed erythroid differentiation, with a marked increase in apoptosis and G_0_/G_1_ cell cycle arrest, and activation of the p53 pathway [11]. *RPS19*-deficient cells exhibit reduced levels of *Cyclin*-*E*, *CDK2* and retinoblastoma (Rb) protein, suggesting cell cycle arrest in the G1 phase. Interestingly, *RPS24*-deficient cells showed increased levels of the cell cycle inhibitor p21 and a seemingly opposing increase in *Cyclin*-*E*, *CDK4* and *CDK6*. The exact mechanism of DBA may be a combined effect of ribosome synthesis and p53 activation, and more efforts are needed to correlate specific mutations to the disease phenotypes such as its impact on erythropoiesis. To achieve this goal, the regulatory networks for each RP mutation may be needed to be established for careful examination and comparison. In addition, unbiased screen approaches are also necessary to understand DBA pathogenesis using various models, such as mouse and zebrafish, in addition to patient samples because no mutations were found in approximately 50% of clinically diagnosed DBA patients.

The ease of maintenance, the large number of offspring, and the possibility to conduct high resolution in vivo imaging have made zebrafish an attractive model organism of choice in biomedical research. While deep sequencing is now widely accepted as a useful tool to investigate human disease mechanisms and gene functions [13, 14]; it has not been widely used in zebrafish for mechanistic studies of hematopoiesis and related diseases [15–18]. In this study, by applying the high-throughput RNA-seq and miRNA-seq methods, we characterized the deregulated RNAs, miRNAs and molecular regulatory networks in *RPS24*-deficient zebrafish embryos in comparison with controls. Several key genes and miRNAs related to hematopoiesis, vascular development and apoptosis were found to be dysregulated in *RPS24*-deficient zebrafish. In addition, several hematopoietic miRNA signatures are uniquely expressed in *RPS24* MO such as miR-142-3p and miR-29a, which have been previously reported to be required for the formation and differentiation of hematopoietic stem cells [19, 20]. We further confirmed that one of the uniquely expressed miRNAs, miR-142-3p, plays a critical role during erythrocyte progenitor cell and HSC formation. A comprehensive regulatory network for *RPS24* MO-specific DBA was constructed and can be used to identify the mechanisms of key miRNAs and gene pathways in this *RPS24* MO DBA Model.

## Results

### Hematopoietic defects in *RPS24*MO

**Figure 1:**
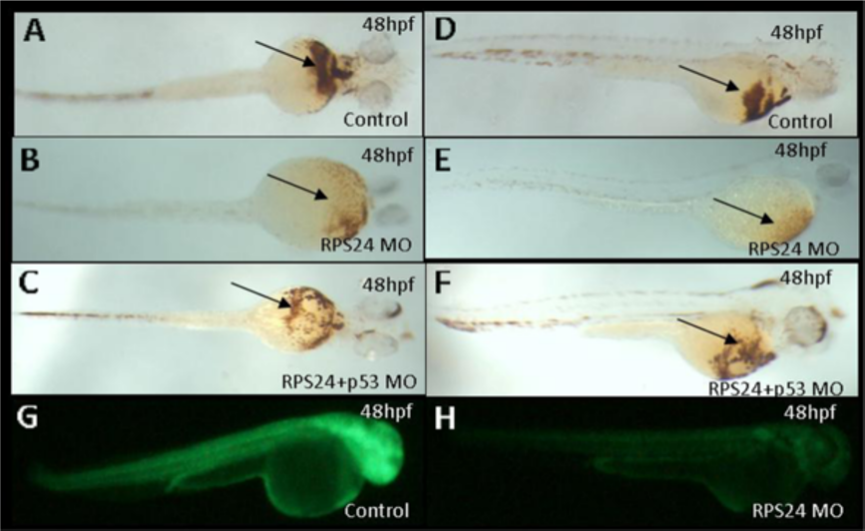
**Hemoglobin staining of embryos injected with rps24 MO using o**-**dianisidine and effectiveness of rps24 MO. (A**-**F)** O-staining of rps24 MO embryos showed a drastic reduction in the number of hemoglobin-stained blood cells when rps24 is knockdown (**A** and **D** are controls, **B** and **E** are rps24 knockdown) and partially rescued phenotype by co-injection of p53 MO (**C** and **F**). **(G**-**H)** The sequence of rps24 MO is a compliment of 1–24 bp of rps24 cDNA. Embryos co-injected with 25 ng rps24: egfp DNA and 5 ng control MO produced green fluorescent protein **(G)**, and the expression of green fluorescent fusion protein was inhibited by co-injection with 2 ng rps24 Mo **(H)**. **A**, **B**, **C** are ventral view; **D**, **E**, **F**, **G** and **H** are lateral view.

**Figure 2:**
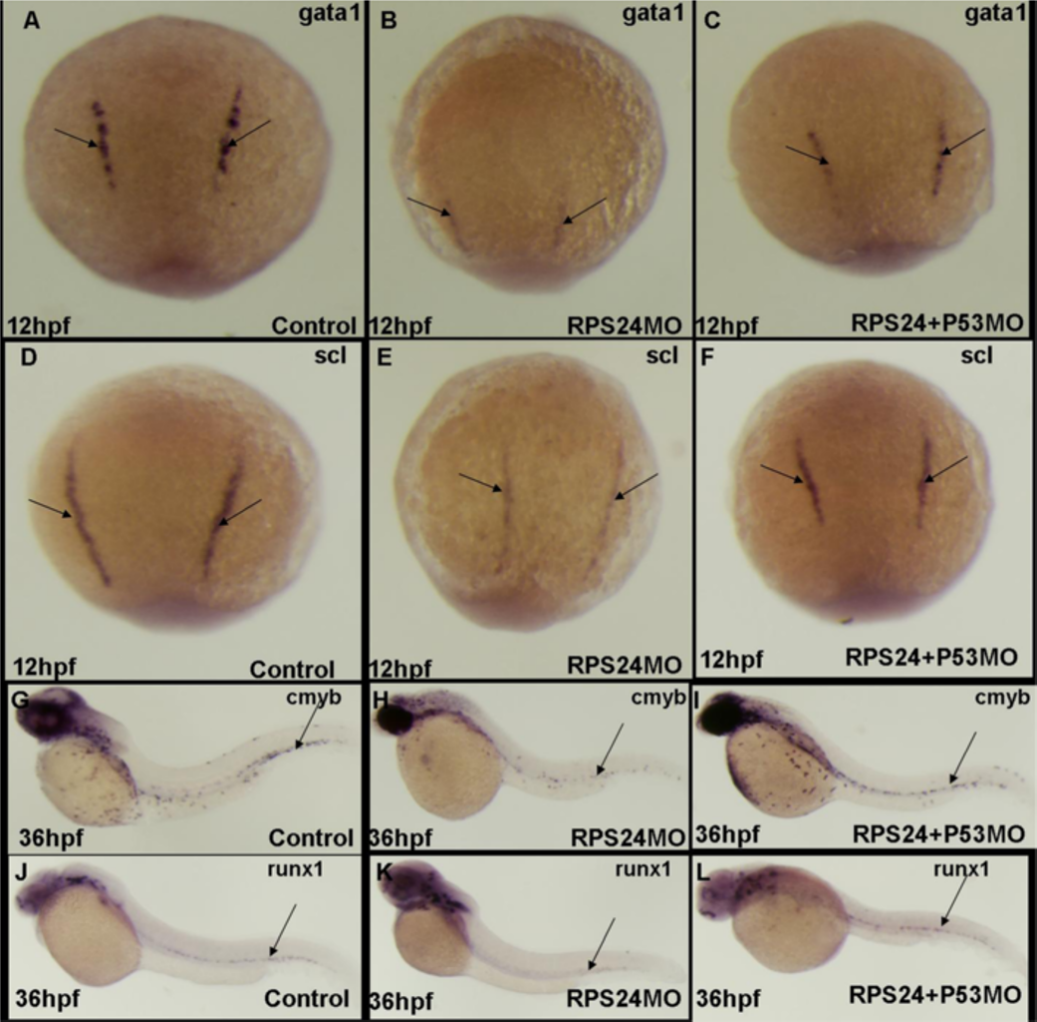
**RPS24 is required for both primitive and definitive hematopoiesis zebrafish partly mediated by p53 pathway. (A**-**C)** The expression of gata1 (black arrow) was significantly decreased in rps24 MO and can be partly rescued in rps24 and p53 double MO at 48 hpf. **(D**-**F)** The expression of scl was significantly decreased in rps24 MO and can be partly rescued in rps24 and p53 double MO at 48 hpf. **(G**-**L)** The expression of cmyb and runx1 was significantly decreased in rps24 MO and can be partly rescued in rps24 and p53 double MO at 48 hpf.

To study the regulatory network and the function of *RPS24* during hematopoiesis in zebrafish, we first established an *RPS24*-deficient zebrafish model using Morpholino knockdown (MO) technology. The embryos injected with control MO did not display any morphological changes; however, the *RPS24* MO exhibited various phenotypes, including tail deformities and hematopoietic defects. The hemoglobin staining results indicated that hemoglobin-stained blood cells were markedly decreased in *RPS24* MO at 48 hpf and were partially rescued in *RPS24* + *p53* MO embryos (Figure 1), similar to our previous *RPS19* MO and *RPL11* MO phenotypes [18]. To further determine if *RPS24* deficiencies in zebrafish cause hematopoietic defects that resemble DBA patients, we analyzed multiple markers of primitive and definitive hematopoiesis by RNA whole-mount *in situ* hybridization. Both the expression of the hemangioblast marker *scl* and primitive erythroid progenitor marker *gata1* were decreased in *RPS24* MO at 12 hpf. Similarly, the expression of the definitive hematopoietic stem cell markers *cmyb* and *runx1* were also decreased significantly in *RPS24* MO at 48 hpf. Furthermore, both primitive and definitive hematopoiesis phenotypes caused by *RPS24* MO were partially rescued by knocking-down *p53*, suggesting that *RPS24* is required for both primitive and definitive hematopoiesis, which are both partially mediated by the p53 pathway (Figure 2).

### Global changes of gene expression in *RPS24*MO

The libraries were sequenced using the Illumina Hi-seq 2000 Genome Analyzer platform with paired-end 100 base-pair tags to a depth of 37–40 million reads. These reads were then mapped to the zebrafish genome assembly 2010 version (Zv9). Approximately 16–17 million reads were mapped to this genome, accounting for 40%-50% of the total reads. More than thirteen thousand genes were detected by RNA-seq, which represented 82% of total genes assembled in Zv9 zebrafish genome (Table 1). Based on the FPKM values, the Cuffdiff package was used for differential expressed gene detection for *RPS24* MO and the control. In total, 448 genes were found to be expressed significantly different in *RPS24* MO when compared to the control (FPKM >1, fold-change >1.3, and p-value <0.01). Majority of these genes (404 out of 448) were down-regulated, which demonstrated that some of biological functions might be repressed due to *RPS24* deficiency.

**Table 1:** **Summary of mRNA**-**seq and miRNA**-**seq data mapping results**

	mRNA-seq		miRNA-seq	
	***RPS24***MO	Control	***RPS24***MO	Control
**Total reads**	37,681,854	39,819,594	10,877,136	3,796,697
**Mapped reads**	16,292,078	17,047,289	1,838,474	965,240
**Percentage of mapped reads**	43%	43%	17%	25%
**Matched genes/** **miRNAs**	13,092	13,205	220	214
**Percentage of matched genes/** **miRNAs**	82%	83%	48%	46%

### GO term enrichment analysis

As few genes were found up-regulated in *RPS24*-deficient zebrafish, there is no GO term significantly enriched by them. However, down-regulated genes, accounting for more than 90% of total deregulated genes, were associated with the development of several systems (skeletal, nervous, and sensory) and organs (heart, eye, and ear) (Additional file 1: Table S1). It indicated that these developmental processes might be affected more by the deregulation of enriched genes. The phenotypic observations of tail deformities of *RPS24*-deficient zebrafish might be one of the outcomes resulting from these abnormalities.

Due to the large numbers of down-regulated genes in *RPS24* MO and a plurality of their enriched GO terms, we were also concerned about the biological functions of down-regulated genes with higher fold-change of the control (fold-change >5, and p-value <0.01). Of particular interest, the genes were enriched in not only transcription, DNA/RNA metabolism, and organ development, but cell adhesion (Additional file 2: Table S2). This observation reveals that these functions may be damaged significantly by *RPS24* deficiency.

### Global changes of hematological gene expression in *RPS24*MO

**Figure 3:**
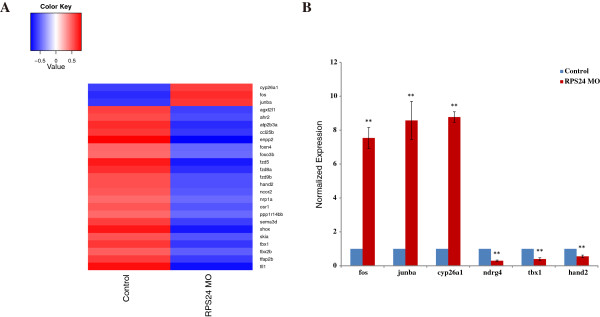
**Differentially expressed hematological genes in** ***RPS24*** **MO. (A)** A total of 25 differentially expressed hematological genes (by fold-change >1.3 and p-value <0.05) were detected in *RPS24*-deficient zebrafish embryos in 48hpf. The expression intensity of these genes were normalized to (-1, 1) and represented as heat map by R script. **(B)** Representative experimental validation of the regulated genes by Real-time PCR analysis. Gene expression was represented as the mean ± SD and One-way ANOVA was performed for comparison between Control and *RPS24*-deficient zebrafish embryos (**P <0.001, *P <0.01, n = 3). Gene expression in Control samples was normalized to 1.

To further dissect the molecular mechanisms for the defect of hematopoiesis due to *RPS24* knockdown, we searched all the related keywords in the Gene Ontology database (http://www.geneontology.org/) and obtained 1259 genes related to the hematological system in the zebrafish genome. Most of the erythroid gene expression was comparable in *RPS24* MO compared to the control; however, 3 up-regulated genes and 22 down-regulated genes (fold-change >1.3 and p-value <0.01) were identified (Figure 3A and Additional file 3: Table S3). Among the three up-regulated genes, *fos* and *junba* were also up-regulated in *RPL11* MO zebrafish and were independent of the p53 pathway regulation [21]. Frizzled homolog family genes (*fzd5*, *fzd8a*, and *fzd9b*), which are important receptors in Wnt signaling and play critical roles during early development process, were repressed in *RPS24* MO [22, 23]. In addition, *fzd5*, *nrp1a*, *sema3d*, and *tbx1* were also significantly down-regulated in *RPS24* MO as in *RPS19* MO zebrafish, and *fzd5* was found to be dependent of the p53 pathway [18]. To confirm the RNA-seq results, we randomly examined 6 of these genes using RT-PCR and we found that the expressional variation trend in RT-PCR results were similar to that identified from the transcriptome analysis. (Figure 3B).

It has been previously demonstrated that genes required for the earlier development of the vascular system may be associated with the initiation of the definitive hematopoietic program [24]. In addition, the depletion of several ribosomal proteins induces apoptosis and leads to a decrease in cell growth of primary hematopoietic cells [11]. To further investigate the regulations of genes participated in these functions including vascular development, cell growth and apoptosis, which are associated with primitive and definitive hematopoiesis, we analyzed the expression of genes associated with these functions. One up-regulated and 29 down-regulated vascular development-related genes were observed, suggesting that the defects may be related to the hematopoietic system in *RPS24* MO (Additional file 4: Table S4). Meanwhile, we found 6 down-regulated genes associated with cell growth, and none were up-regulated (Additional file 5: Table S5). In *RPS24* MO zebrafish, 8 genes with roles in apoptosis were expressed much lower, and one gene was expressed higher than in the control (Additional file 6: Table S6).

### The miRNA signatures of *RPS24*deficiency zebrafish

**Figure 4:**
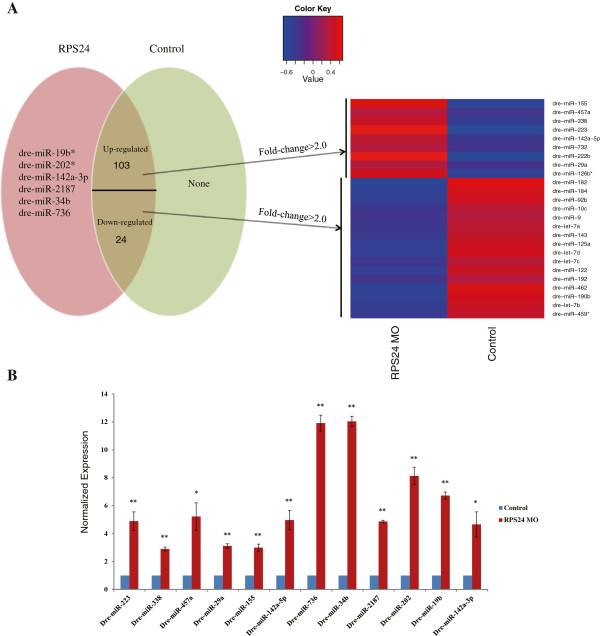
**Differentially regulated known miRNAs. (A)** Specific expressed miRNAs in *RPS24* and Control are represented in the form of Venn diagrams. A subset of miRNAs that are differentially regulated by fold-change >2.0 and p-value <0.05 is in the overlapped area and their normalized expression levels can be seen in the heat map. **(B)** Representative experimental validation of the regulated miRNAs by Real-time PCR analysis. Gene expression was represented as the mean ± SD and One-way ANOVA was performed for comparison between Control and *RPS24*-deficient zebrafish embryos (**P <0.001, *P <0.01, n = 3). Gene expression in Control samples was normalized to 1.

miRNA-seq libraries were constructed and sequenced using the Illumina Hi-seq 2000 Genome Analyzer platform with 80 base-pair tags to a depth of 3.8-10.9 million reads. These reads were mapped to the miRBase to detect known miRNAs. Approximately 0.9-1.8 million reads were mapped to miRNAs in the miRBase (Table 1). The miRNA expression patterns were compared in *RPS24 MO* with their control counterparts. A total of 103 up-regulated miRNAs and 24 down-regulated miRNAs were identified (p-value <0.05). Among these miRNAs, 6 miRNAs were highly expressed in *RPS24* MO but were barely detected in control embryos (Figure 4A). We further confirmed a total of 12 regulated miRNAs by performing RT-PCR. The miRNA-seq results were validated by specific PCR with significant differences (p-value < 0.01) between *RPS24*-deficient zebrafish embryos and the controls (Figure 4B).

**Figure 5:**
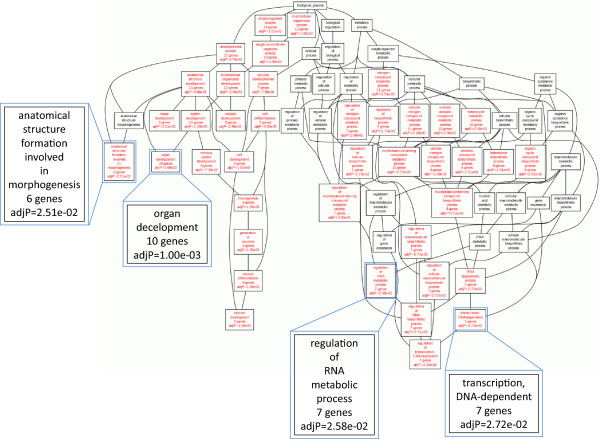
**Gene ontology analysis of overlapped genes in up**-**regulated**-**miRNA targets set and down**-**regulated genes set.** The enriched terms (statistical significance p <0.01) are shown in red.

To observe the biological functions affected by regulatory miRNAs, we used the MicroCosm Targets database (http://www.ebi.ac.uk/enright-srv/microcosm/htdocs/targets/v5/) for miRNA target prediction and DAVID for gene functional annotation [25]. We obtained 3900 potential target genes of 9 significantly up-regulated miRNAs (fold-change >2.0 and p-value <0.05) and 6735 potential target genes of 16 significantly down-regulated miRNAs (fold-change >2.0 and p-value <0.05). Multiple false positive results occur in gene prediction, and the targets of certain miRNAs may be altered in different cellular environments; therefore, we overlapped the prediction results with the mRNA-seq results. We found 8 up-regulated genes that were potentially regulated by lower expressed miRNAs and 36 down-regulated genes that were potentially regulated by higher expressed miRNAs. These genes were associated with the development and morphogenesis of organs and systems (especially nervous system) (Figure 5). Generally, these results were in agreement with the functional analysis of the studied regulated genes, which suggested that these miRNAs may also be the causes of these abnormalities.

### The regulatory network constructed by regulated genes and miRNAs in *RPS24*MO

**Figure 6:**
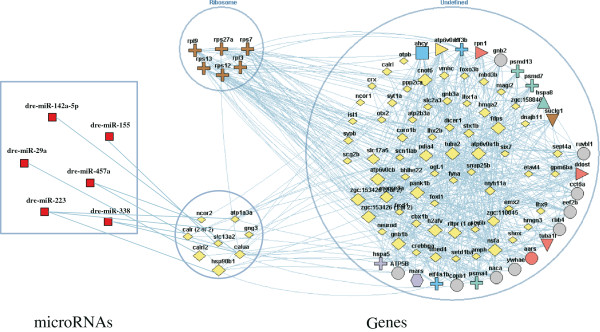
**Network analysis of differential expressed genes and miRNAs.** The yellow diamond represents the down-regulated genes (FPKM >1, fold-change >1.3, p-value <0.01), and red square represents the up-regulated miRNAs (fold-change >2.0, p-value <0.05). Other nodes are connected with our query genes by 1-expansion-depth. All the lines represent direct correlation of nodes. The size of nodes represents its connectivity. Bigger the node is, larger its connectivity is. Central nodes (connectivity >30) are *atp6v0a1b*, *pank1b*, *fdps*, *h2afv*, *atp6v0cb*, *cnot6*, *nsfa*, *gnb1b*, and *zgc*:*153426*.

To understand the interacting relationships of important regulated genes, the deregulated genes were entered into the FunCoup framework (http://funcoup.sbc.su.se/search/) to deduce genome-wide functional couplings by 1-expansion-depth (Figure 6). We found that ribosomal proteins such as *rpl3*, *rps7*, *rpl9*, *rps12*, *rps13*, and *rps27a* showed important roles in this network. Moreover, *atp6v0a1b*, *pank1b*, *fdps*, *h2afv*, *atp6v0cb*, *cnot6*, *nsfa*, *gnb1b*, and *zgc*:*153426* (connectivity >30) presented in the network as central nodes(Additional file 7: Table S7). *fdps* has been previously suggested as an enzyme that regulates arteriovenous angiogenesis by targeting the HMG-CoA reductase (HMGCR) pathway [26]. *h2afv* has been demonstrated as an important regulator in hematopoietic stem cells [27]. The dysregulation of these central nodes playing important roles in the hematological system may be a key step in causing defective hematopoiesis. Except for hematopoiesis, several central nodes participate in the developmental process. For example, *atp6v0cb* plays a critical role during vertebrate eye development and neuronal differentiation, *nsfa* is essential for the organization of myelinated axons, and *gnb1b* is associated with neurogenesis in the zebrafish retina [28–32]. These findings may explain the developmental abnormalities of *RPS24*-deficient zebrafish.

We then constructed the targeting relationships between significantly regulated miRNAs and the differentially expressed genes using MicroCosm Targets (Figure 6). In total, 6 up-regulated miRNAs (such as dre-miR-142a-5p, dre-miR-155, dre-miR-29a, dre-miR-457a, dre-miR-223, and dre-miR-338) were joined to the gene regulatory network by potentially targeting relationships with 8 down-regulated genes. This network provides us with an entire picture of interactions of regulatory genes and miRNAs in *RPS24*-deficient zebrafish, which may explain why hematopoiesis is defective and development is abnormal.

### miR-142-3p is required for primitive erythroid progenitor cell and HSC formation

**Figure 7:**
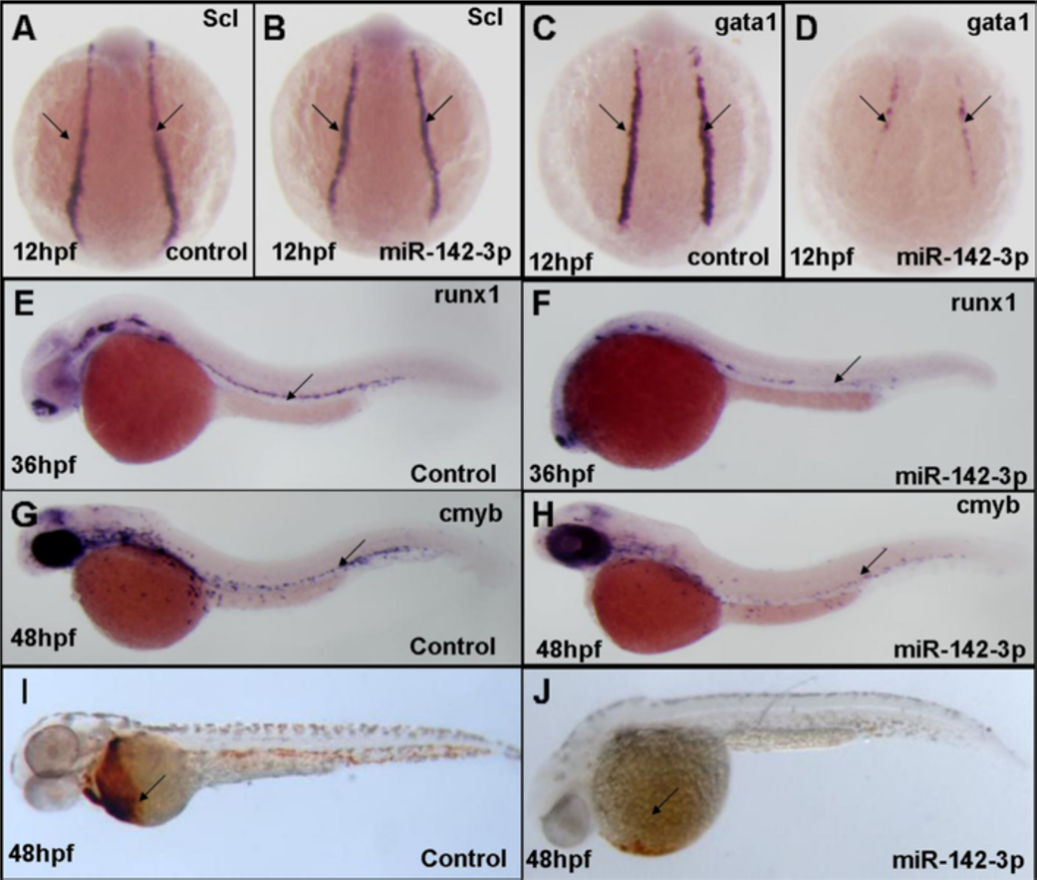
**Enforced expression of miR**-**142**-**3p suppresses the primitive erythrocyte progenitors and HSCs formation. (A**-**B)** the expression of scl is comparable; **(C**-**D)** the expression of gata1 is significantly decreased; **(E**-**H)** the expression of cmyb and runx1 is significantly repressed; **(I**-**J)** the number of hemoglobin-stained blood cells shows a drastic reduction when miR-142-3p is ectopically expressed (compared with controls).

To study the hematopoietic phenotype caused by increased miR-142-3p signaling, we overexpressed miR-142-3p using miR-142-3p duplex overexpression. We observed that embryos have much fewer circulating blood cells although their heart and vessel formation are normal. Using O-dianisidine hemoglobin staining, a significant decrease of mature erythroid cells was detected when miR-142-3p was overexpressed. However, the *scl* expression was comparable to the control embryos, suggesting that miR-142-3p is not required for early hemangioblast formation. Interestingly, the *gata1*, *cmyb* and *runx1* expression were all markedly decreased when miR-142-3p was ectopically expressed (Figure 7), indicating that miR-142-3p affects HSC formation in zebrafish. Furthermore, all of the above hematopoietic phenotypes were alleviated by co-injection of miR-142-3p mimics and MO (data not shown). Overall, these data suggest that increased miR-142-3p represses primitive erythroid progenitor cell and HSC formation.

## Discussion

The hallmark of DBA syndrome in patients is severe macrocytic anemia during early life and physical deformities that are frequently associated with the disease with no explanations for the heterogeneity of the clinical manifestations [5, 12]. Approximately 50% of DBA patients showed autosomal dominant ribosomal gene mutations such as *RPS19*, *RPS24*, *RPL11*, *RPL5* and others that leads to impaired ribosome biogenesis, increased apoptosis, and defective hematopoiesis. While zebrafish has been increasingly used as a model system for hematological disorders including DBA studies, systemic analysis of the effects of individual RP genes on the transcriptome regulatory network in zebrafish has not been reported [18, 33]. In this study, we first established *RPS24* deficiency model in zebrafish and found phenotypes resembling DBA, including impaired ribosome biogenesis, increased apoptosis, and defective hematopoiesis, similar to the phenotypes of *RPS19* MO and *RPL11* MO [18, 34]. Further transcriptome analysis indicates that *RPS24* is required for both primitive and definitive hematopoiesis partially via *p53* pathway, *RPS19* is dispensable for definitive hematopoiesis and *RPL11* is required for primitive hematopoiesis independent of p53 pathway [18, 34]. It has also recently been reported that the two RP paralogs, *RPL22* and *RPL22*-like 1 proteins, play distinct and antagonistic roles during hematopoietic stem cell development [35]. These results from various groups suggest that while ribosomal gene mutations lead to similar phenotypes, the underlying molecular mechanism for the DBA may be quite different and a reflection of the remarkable complexity of DBA.

In *RPS24*-deficient embryos, genes enriched in GO categories of regulation of transcription, regulation of RNA metabolic process, and development and differentiation of organs and systems were significantly down-regulated, suggesting that the phenotypic traits containing tail deformities and hematopoietic defects are only parts of defects due to *RPS24* deficiency. The damages of deficiency are not limited to what we observed before, but are also involved in some basic biological processes such as transcription and RNA metabolic process, and development and differentiation of other organs and tissues, such as heart, neuron, sensory organ, and skeletal system. It demonstrates that *RPS24* is vitally important for the whole organism development and biological processes maintaining. It is noteworthy that transcriptome of *RPS24*-deficient zebrafish model doesn’t indicate the defective translation processes, although RPS24 is one of the ingredients of ribosomes which catalyze mRNA translation and protein synthesis. However, genes involved in regulation of RNA transcription and RNA metabolic process are significantly down-regulated by RPS24 deficiency. It suggests that RPS24 depletion may significantly affect the transcription process rather than the translation process. Moreover, we noticed that the dramatically down-regulated genes (fold-change >5) were also associated with processes of cell adhesion and biological adhesion, except for above terms. Cell adhesion has been found to play a key role in vascular pathology, such as in sickle cell anemia [36]. Thus, cell adhesion, which may be severely affected in *RPS24*-deficient zebrafish due to the significantly reduced expression of the involved genes, may be the common alteration of anemia diseases.The defects caused by the alterations of gene expression may ultimately contribute to hematopoietic pathology.

Central genes are defined as nodes with connectivity more than 30. Because the central nodes potentially interact with much more regulatory genes than others, they may be the key regulators in RPS24-deficient zebrafish embryos. Among the central node genes that we identified in the regulatory network analysis, such as *atp6v0a1b*, *pank1b*, *fdps*, *h2afv*, *atp6v0cb*, *cnot6*, *nsfa*, *gnb1b* and *zgc*:*153426*, *h2afv* was demonstrated to be a regulator in hematopoietic stem cells [27]. We found that *h2afv* interacts with many more genes in the network as well as other central node genes and thus may have more significant effects on the *RPS24*-deficient defects. While the molecular mechanisms as how the products of these gene lead to DBA still needs to be further investigated. Furthermore, we identified additional genes associated with erythropoiesis and hematopoiesis defects that are also identified in *RPL11*- and *RPS19*-deficient zebrafish, thus confirming their key roles during hematological development [18].

In addition to the extraordinary accuracy of the global view of expression levels, high sensibility is also an advantage of deep sequencing for the analysis of miRNA that expressed at low abundance in cells. In this study, miRNome analysis, coordinated and complemented with transcriptome analysis, also provides us some clues to the molecular mechanism of defective hematopoiesis caused by *RPS24* deficiency. *miR*-*155* and *miR*-*223*, which are significantly up-regulated in *RPS24* MO, are highly specific for hematopoietic cells in mouse and human [37], suggesting that they might be crucial factors in the hematopoietic pathogenesis of *RPS24*-deficiency-induced DBA. Furthermore, 6 highly expressed miRNAs in *RPS24* MO were barely detected in control embryos. Among them, *dre*-*miR*-*34b* was genetically upstream of *cmyb*, whose expression marked appearance of nascent HSCs in the zebrafish. *Dre*-*mir*-*142a*-*3p* was demonstrated as a hematopoietic-specific miRNA required for the definitive hemangioblast formation, HSCs formation and subsequent differentiation [19, 20]. There are several targets of it that are known to be involved in hematopoiesis, such as irf7, TGFβr1 and et al. [19, 20]. We further found that enforced *miR*-*142*-*3p* expression could inhibit the formation of primitive erythrocyte progenitor and HSCs formation. It suggests that either gain or loss of function of *miR*-*142*-*3p* leads to severe defects of HSCs formation and that *miR*-*142*-*3p* is an essential regulator for primitive and definitive hematopoiesis. Further studies are required to fully elucidate the genetic mechanisms of how *RPS24* regulates both the miRNAs that are responsible for the initiation of definitive hemangioblast specification and their targets.

## Conclusions

Except for the genes previously confirmed as important regulators in hematopoietic stem cells, several novel key genes and miRNAs were found to have interactions with other genes in the network, thus the current study highlights the use of genome-wide mRNA and miRNA expression analysis to reveal new links in the genes and miRNAs regulatory network at the transcriptome level. Several key genes and miRNAs were found to have interactions with other genes in the network (Figure 6). Some of the genes were confirmed as important regulators in hematopoietic stem cells and other cells [28]. This study thus provides a whole picture of expression changes and regulated relations of genome-wide transcripts. Collective analysis of similar studies would help to truly dissect the underlying molecular mechanisms of the heterogeneous phenotypes in DBA patients with similar or variable gene mutations. It will also help the discovery process of novel key genes and miRNAs and the design of novel drug targets.

## Methods

### Zebrafish care, MO injection and O-dianisidine staining

Wild-type zebrafish (*Danio rerio*; AB type) were raised under standard library conditions, and embryo stages were determined as previously described [38, 39]. *RPS24* Morpholino (5- TGACAGTGACTGTGTCGTTCATCTT-3), *RPS24* control MO (5-TGAAAGTAACTGTGACGGTCGTATT-3), *miR*-*142*-*3p* Morpholino (5-TCCATAAAGTAGGAAACACTACACT-3), *miR*-*142*-*3p* control Mo (5-TCaAaAAAtTAcGAAACgCTACACT-3) and *p53* Morpholino (5- GCGCCATTGCTTTGCAAGAATTG-3) were obtained from Gene-Tools, LLC (Philomath, OR, USA). Based on our initial trials, 0.5 ng of *RPS24* MO and control MO, 20 umol/L miR-142-3p duplexes (Ribo Company, Guangzhou, China), 2 ng miR-142-3p MO and control MO in Danieau’s buffer were chosen as the optimal concentration, which showed significant decrease in O-staining signal, but no obvious morphological defects when compared with the control embryos. The O-dianisidine (Sigma-Aldrich Company, Saint Louis, USA) staining protocols were as previously described [18, 40]. All the studies of zebrafish were approved by the Animal Care and Use Committee of Huazhong University of Science and Technology.

### RNA preparation, library preparation and sequencing

Approximately 50 zebrafish embryos at 48 hpf from different experimental replicates were snap-frozen in liquid nitrogen. The total RNA was extracted using TRIzol (Invitrogen, Carlsbad, California, USA) according to the manufacturer’s instructions. The integrity of RNA samples was determined using 1.2% agarose gel electrophoresis, followed by removal of the residual genomic DNA with RNase-free DNaseI (Ambion, Austin, Texas, USA). mRNA and small RNA libraries were constructed using the Illumina mRNA-Seq and miRNA-seq library preparation kits, respectively. The size distribution and concentration of the libraries were determined by Agilent Bioanalyzer DNA 2000 chip (Agilent Technologies, Santa Clara, California, USA) followed by sequencing on the Illumina Hiseq 2000 Genome Analyzer platform. The RNA-Seq library was sequenced with 2 × 100 bp in pair-end mode by 100-bp lengths, and the miRNA library was sequenced in single-end mode by 80-bp lengths.

### RNA-Seq data analysis

The reads were processed and aligned to the UCSC zebrafish reference genome (build Zv9/danRer7, Jul. 2010) using TopHat (version 1.3.3) [41]. TopHat incorporates the Bowtie v0.12.7 algorithm to perform alignments. TopHat-aligned read files were then entered into Cufflinks (version 1.2.1) software for further analyses, including transcript assembly, abundance estimation, and differential expression and regulation testing in RNA-Seq samples [42]. To calculate gene expression intensity, read counts were normalized to the number of fragments per kilobase of transcript per million mapped reads (FPKM) according to the gene length and total mapped reads [41]. Confidence intervals for estimates of FPKM were calculated using the Bayesian inference method [43]. Cuffdiff then performed the differential expression tests at the level of transcripts, primary transcripts and genes [44]. The genes with FPKM less than 1 were removed from analyses. Differential expressed genes were characterized according to the criterion of fold-change >1.3 and p-value <0.01.

### miRNA-seq data analysis

The FASTX-Toolkit clipper was used to remove sequencing adapters. The .fastq file was then converted to a tab-delimited file which held only the unique sequence read (tag) and its corresponding number of copies. After preprocessing these data, the files were uploaded to DSAP (http://dsap.cgu.edu.tw/index.htm) for clustering of the tags and the classification of non-coding small RNAs and miRNAs based on a sequencing homology search against the Rfam and miRBase database, respectively [45]. The differential-expressed miRNAs were detected by R package DEGseq using the output data of DSAP.

### Gene ontology analysis and network construction

DAVID tools (http://david.abcc.ncifcrf.gov/) were used to identify enriched biological themes and functional-related gene groups [25, 46, 47]. The differentially expressed genes were used for functional annotation analysis against a background gene set containing all the expressed genes. GO enrichment results were accepted with a threshold of Gene-Count ≥5 and P-Value <0.05. The interacting network was constructed for differential expressed genes by FunCoup (http://funcoup.sbc.su.se/search/) [48]. The construction of the linkages between genes and miRNAs was based on the targeting information from MicroCosm Target database (http://www.ebi.ac.uk/enright-srv/microcosm/htdocs/targets/v5/). The importance of nodes in networks was measured on the basis of their connectivity, and the core molecules of networks were considered as nodes that are connected with many more edges.

### Quantitative Real-time PCR

Real-time PCR of mRNA and miRNA was performed using SYBR Green PCR Master Mix (Fermentas, Guangzhou, China) and All-in-One™ miRNA qPCR Kit (GeneCopoeia, Maryland, USA) respectively, according to the manufacturer’s instructions. The experiments were repeated at least in triplicates. The primers for Real-time PCR are shown in Additional file 8: Table S8.

### Whole mount in situ hybridization

Digoxigenin-labeled antisense riboprobes were transcribed from a linearized plasmid containing *gata1*, *scl*, *cmyb and runx1* using DIG RNA labeling Mix and T7 RNA polymerase (Roche, USA) [35, 49]. Whole mount in situ hybridization was conducted as previously described [18, 50].

### Data access

The raw sequence data are available in the Gene Expression Omnibus. The accession number is GSE54270. Although the data remain in private status: http://www.ncbi.nlm.nih.gov/geo/query/acc.cgi?token=ynkbuigknbsrvkf&acc=GSE54270, all the genomic resources generated in this study are provided as Supplementary data sets.

## Electronic supplementary material

Additional file 1: Table S1: Enriched GO biological process terms (Count≥5 and P-Value <0.05) for down-regulated genes (fold-change >1.3 and p-value <0.01) of *RPS24* MO. (DOC 46 KB)

Additional file 2: Table S2: Enriched GO biological process terms (Count ≥5 and P-Value <0.05) for dramatic down-regulated genes (fold-change >5, and p-value <0.01) of *RPS24* MO. (DOC 29 KB)

Additional file 3: Table S3: Differential expressed genes associated with hematological system. (DOC 40 KB)

Additional file 4: Table S4: Differential expressed genes associated with vascular development. (DOC 45 KB)

Additional file 5: Table S5: Differential expressed genes associated with cell growth. (DOC 30 KB)

Additional file 6: Table S6: Differential expressed genes associated with apoptosis. (DOC 30 KB)

Additional file 7: Table S7: Central nodes of the regulatory network constructed by regulated genes and miRNAs in *RPS24* MO. (DOC 31 KB)

Additional file 8: Table S8: The primers designed for Real-time PCR. (DOC 32 KB)

## Abbreviations

DBA: Diamond–Blackfan anemia
RPS24: Ribosomal protein S24
HSC: Hematopoietic stem cells
RPs: Ribosomal proteins.
