# Supplementary material for: Systematic transcriptome analysis of the zebrafish model of diamond-blackfan anemia induced by RPS24 deficiency
Source: BMC Genomics. 2014 Sep 4;15(1):759. doi: 10.1186/1471-2164-15-759 (PMC4169864; doi:10.1186/1471-2164-15-759)
Supplement: Supplementary file 1 — Additional file 1: Table S1: Enriched GO biological process terms (Count≥5 and P-Value <0.05) for down-regulated genes (fold-change >1.3 and p-value <0.01) of RPS24 MO. (DOC 46 KB) [file 12864_2014_6455_MOESM1_ESM.doc]

**Additional file 1: Table S1 Enriched GO biological process terms (Count≥5 and P-Value <0.05) for down-regulated genes (fold-change >1.3 and p-value <0.01) of *RPS24*** MO

| **Term** | **Count** | **%** | **P-Value** |
| --- | --- | --- | --- |
| regulation of transcription | 64 | 19.9 | 6.60E-10 |
| regulation of transcription, DNA-dependent | 49 | 15.2 | 1.40E-07 |
| regulation of RNA metabolic process | 49 | 15.2 | 1.80E-07 |
| neuron differentiation | 15 | 4.7 | 1.10E-04 |
| heart development | 14 | 4.3 | 2.20E-04 |
| sensory organ development | 16 | 5.0 | 1.50E-03 |
| skeletal system development | 9 | 2.8 | 2.70E-03 |
| biological adhesion | 14 | 4.3 | 3.20E-03 |
| cell adhesion | 14 | 4.3 | 3.20E-03 |
| eye morphogenesis | 8 | 2.5 | 4.00E-03 |
| neuron development | 9 | 2.8 | 6.50E-03 |
| embryonic organ development | 12 | 3.7 | 7.60E-03 |
| regulation of cell development | 6 | 1.9 | 8.80E-03 |
| tube development | 9 | 2.8 | 9.90E-03 |
| embryonic morphogenesis | 15 | 4.7 | 1.10E-02 |
| embryonic heart tube development | 6 | 1.9 | 1.10E-02 |
| eye development | 11 | 3.4 | 1.30E-02 |
| embryonic organ morphogenesis | 8 | 2.5 | 1.80E-02 |
| transcription | 24 | 7.5 | 2.10E-02 |
| cell morphogenesis involved in differentiation | 7 | 2.2 | 2.30E-02 |
| cartilage development | 6 | 1.9 | 2.30E-02 |
| cell fate commitment | 6 | 1.9 | 2.50E-02 |
| camera-type eye morphogenesis | 5 | 1.6 | 3.00E-02 |
| regulation of neurogenesis | 5 | 1.6 | 3.30E-02 |
| regulation of nervous system development | 5 | 1.6 | 3.30E-02 |
| inner ear morphogenesis | 5 | 1.6 | 3.30E-02 |
| axonogenesis | 6 | 1.9 | 3.60E-02 |
| cell morphogenesis involved in neuron differentiation | 6 | 1.9 | 3.60E-02 |
| ear morphogenesis | 5 | 1.6 | 3.60E-02 |
| neuron projection morphogenesis | 6 | 1.9 | 3.90E-02 |
| neurological system process | 7 | 2.2 | 4.00E-02 |
| neuron projection development | 6 | 1.9 | 4.70E-02 |
