# Supplementary material for: Systematic transcriptome analysis of the zebrafish model of diamond-blackfan anemia induced by RPS24 deficiency
Source: BMC Genomics. 2014 Sep 4;15(1):759. doi: 10.1186/1471-2164-15-759 (PMC4169864; doi:10.1186/1471-2164-15-759)
Supplement: Supplementary file 2 — Additional file 2: Table S2: Enriched GO biological process terms (Count ≥5 and P-Value <0.05) for dramatic down-regulated genes (fold-change >5, and p-value <0.01) of RPS24 MO. (DOC 29 KB) [file 12864_2014_6455_MOESM2_ESM.doc]

**Additional file 2: Table S2 Enriched GO biological process terms (Count ≥5 and P-Value <0.05) for dramatic down-regulated genes (fold-change >5, and p-value <0.01) of *RPS24*** MO

| **Term** | **Count** | **%** | **P-Value** |
| --- | --- | --- | --- |
| regulation of transcription | 23 | 22.1 | 5.00E-05 |
| regulation of transcription, DNA-dependent | 18 | 17.3 | 4.10E-04 |
| regulation of RNA metabolic process | 18 | 17.3 | 4.50E-04 |
| cell adhesion | 6 | 5.8 | 2.90E-02 |
| biological adhesion | 6 | 5.8 | 2.90E-02 |
| sensory organ development | 6 | 5.8 | 4.90E-02 |
