# Supplementary material for: Systematic transcriptome analysis of the zebrafish model of diamond-blackfan anemia induced by RPS24 deficiency
Source: BMC Genomics. 2014 Sep 4;15(1):759. doi: 10.1186/1471-2164-15-759 (PMC4169864; doi:10.1186/1471-2164-15-759)
Supplement: Supplementary file 3 — Additional file 3: Table S3: Differential expressed genes associated with hematological system. (DOC 40 KB) [file 12864_2014_6455_MOESM3_ESM.doc]

**Additional file 3:** Table S3 Differential expressed genes associated with hematological system

| **Gene** | **Regulation** | **Fold Change** | **p-Value** | **Description** |
| --- | --- | --- | --- | --- |
| cyp26a1 | up | 4.03 | 8.49E-06 | cytochrome P450, subfamily XXVIA, polypeptide 1 |
| fos | up | 4.95 | 3.06E-07 | v-fos FBJ murine osteosarcoma viral oncogene homolog |
| junba | up | 4.36 | 2.66E-06 | jun B proto-oncogene a |
| agxt2l1 | down | 0.25 | 3.25E-05 | alanine-glyoxylate aminotransferase 2-like 1 |
| ahr2 | down | 0.28 | 2.48E-05 | aryl hydrocarbon receptor 2 |
| atp2b3a | down | 0.20 | 5.91E-05 | ATPase, Ca++ transporting, plasma membrane 3a |
| ccl25b | down | 0.23 | 2.57E-04 | chemokine (C-C motif) ligand 25b |
| enpp2 | down | 0.11 | 3.87E-04 | ectonucleotide pyrophosphatase/phosphodiesterase 2 |
| foxn4 | down | 0.35 | 5.54E-04 | forkhead box N4 |
| foxo3b | down | 0.37 | 1.01E-03 | forkhead box O3b |
| fzd5 | down | 0.16 | 1.59E-05 | frizzled homolog 5 |
| fzd8a | down | 0.21 | 3.16E-06 | frizzled homolog 8a |
| fzd9b | down | 0.29 | 6.59E-04 | frizzled homolog 9b |
| hand2 | down | 0.29 | 1.18E-04 | heart and neural crest derivatives expressed transcript 2 |
| ncor2 | down | 0.31 | 1.23E-04 | nuclear receptor co-repressor 2 |
| nrp1a | down | 0.36 | 2.42E-06 | neuropilin 1a |
| osr1 | down | 0.31 | 5.63E-04 | odd-skipped related 1 (Drosophila) |
| ppp1r14bb | down | 0.36 | 9.80E-04 | protein phosphatase 1, regulatory (inhibitor) subunit 14Bb |
| sema3d | down | 0.25 | 3.92E-05 | semaphorin 3d |
| shox | down | 0.16 | 1.13E-04 | short stature homeobox |
| skia | down | 0.30 | 1.22E-04 | nuclear oncoprotein skia |
| tbx1 | down | 0.23 | 1.30E-05 | T-box 1 |
| tbx2b | down | 0.33 | 3.85E-04 | T-box 2b |
| tfap2b | down | 0.24 | 4.72E-08 | transcription factor AP-2 beta |
| tll1 | down | 0.14 | 9.14E-04 | tolloid-like 1 |
