# Supplementary material for: Systematic transcriptome analysis of the zebrafish model of diamond-blackfan anemia induced by RPS24 deficiency
Source: BMC Genomics. 2014 Sep 4;15(1):759. doi: 10.1186/1471-2164-15-759 (PMC4169864; doi:10.1186/1471-2164-15-759)
Supplement: Supplementary file 4 — Additional file 4: Table S4: Differential expressed genes associated with vascular development. (DOC 45 KB) [file 12864_2014_6455_MOESM4_ESM.doc]

**Additional file 4:** Table S4 Differential expressed genes associated with vascular development

| **Gene** | **Regulation** | **Fold Change** | **p-value** | **Description** |
| --- | --- | --- | --- | --- |
| cyp26a1 | up | 4.03 | 8.49E-06 | cytochrome P450, subfamily XXVIA, polypeptide 1 |
| apc | down | 0.27 | 1.91E-05 | adenomatosis polyposis coli |
| aplnrb | down | 0.31 | 8.53E-04 | apelin receptor b |
| atp1b2b | down | 0.07 | 9.91E-06 | ATPase, Na+/K+ transporting, beta 2b polypeptide |
| bmpr1aa | down | 0.35 | 6.83E-04 | bone morphogenetic protein receptor, type 1aa |
| enpp2 | down | 0.11 | 3.87E-04 | ectonucleotide pyrophosphatase/phosphodiesterase 2 |
| fgfr2 | down | 0.39 | 3.31E-10 | fibroblast growth factor receptor 2 |
| fzd5 | down | 0.16 | 1.59E-05 | frizzled homolog 5 |
| fzd8a | down | 0.21 | 3.16E-06 | frizzled homolog 8a |
| fzd9b | down | 0.29 | 6.59E-04 | frizzled homolog 9b |
| hand2 | down | 0.29 | 1.18E-04 | heart and neural crest derivatives expressed transcript 2 |
| igf1ra | down | 0.25 | 7.76E-04 | insulin-like growth factor 1a receptor |
| isl1 | down | 0.25 | 2.84E-05 | islet1 |
| ndrg4 | down | 0.19 | 4.22E-07 | N-myc downstream regulated gene 4 |
| nipbla | down | 0.28 | 5.19E-05 | nipped-b homolog a (Drosophila) |
| notch2 | down | 0.37 | 9.02E-04 | notch homolog 2 |
| nr2f2 | down | 0.30 | 8.13E-05 | nuclear receptor subfamily 2, group F, member 2 |
| nrp1a | down | 0.36 | 2.42E-06 | neuropilin 1a |
| osr1 | down | 0.31 | 5.63E-04 | odd-skipped related 1 (Drosophila) |
| sema3d | down | 0.25 | 3.92E-05 | semaphorin 3d |
| sfrp1a | down | 0.24 | 9.28E-05 | secreted frizzled-related protein 1a |
| sfrp2 | down | 0.20 | 2.99E-07 | secreted frizzled-related protein 2 |
| shox2 | down | 0.18 | 1.47E-04 | short stature homeobox 2 |
| skia | down | 0.30 | 1.22E-04 | nuclear oncoprotein skia |
| tbx1 | down | 0.23 | 1.30E-05 | T-box 1 |
| tbx2a | down | 0.25 | 2.14E-05 | T-box gene 2a |
| tbx2b | down | 0.33 | 3.85E-04 | T-box 2b |
| tfap2b | down | 0.24 | 4.72E-08 | transcription factor AP-2 beta |
| tll1 | down | 0.14 | 9.14E-04 | tolloid-like 1 |
| vmhc | down | 0.07 | 3.81E-10 | ventricular myosin heavy chain |
