# Supplementary material for: Systematic transcriptome analysis of the zebrafish model of diamond-blackfan anemia induced by RPS24 deficiency
Source: BMC Genomics. 2014 Sep 4;15(1):759. doi: 10.1186/1471-2164-15-759 (PMC4169864; doi:10.1186/1471-2164-15-759)
Supplement: Supplementary file 5 — Additional file 5: Table S5: Differential expressed genes associated with cell growth. (DOC 30 KB) [file 12864_2014_6455_MOESM5_ESM.doc]

**Additional file 5:** Table S5 Differential expressed genes associated with cell growth

| **Gene** | **Regulation** | **Fold Change** | **p-value** | **Description** |
| --- | --- | --- | --- | --- |
| epha4b | down | 0.18 | 2.34E-04 | eph receptor A4b |
| foxd1 | down | 0.21 | 1.46E-05 | forkhead box D1 |
| foxn4 | down | 0.35 | 5.54E-04 | forkhead box N4 |
| nrp1a | down | 0.36 | 2.42E-06 | neuropilin 1a |
| robo2 | down | 0.29 | 1.20E-04 | roundabout homolog 2 |
| sema3d | down | 0.25 | 3.92E-05 | semaphorin 3d |
