# Supplementary material for: Systematic transcriptome analysis of the zebrafish model of diamond-blackfan anemia induced by RPS24 deficiency
Source: BMC Genomics. 2014 Sep 4;15(1):759. doi: 10.1186/1471-2164-15-759 (PMC4169864; doi:10.1186/1471-2164-15-759)
Supplement: Supplementary file 6 — Additional file 6: Table S6: Differential expressed genes associated with apoptosis. (DOC 30 KB) [file 12864_2014_6455_MOESM6_ESM.doc]

**Additional file 6:** Table S6 Differential expressed genes associated with apoptosis

| **Gene** | **Regulation** | **Fold Change** | **p-value** | **Description** |
| --- | --- | --- | --- | --- |
| foxq1a | up | 4.07 | 9.26E-05 | forkhead box Q1a |
| apc | down | 0.27 | 1.91E-05 | adenomatosis polyposis coli |
| casp3a | down | 0.36 | 7.89E-04 | caspase 3, apoptosis-related cysteine protease a |
| dlg5a | down | 0.29 | 8.85E-04 | discs, large homolog 5a (Drosophila) |
| fgfr2 | down | 0.39 | 3.31E-10 | fibroblast growth factor receptor 2 |
| foxo3b | down | 0.37 | 1.01E-03 | forkhead box O3b |
| prl | down | 0.17 | 5.81E-04 | prolactin |
| skia | down | 0.30 | 1.22E-04 | nuclear oncoprotein skia |
| tfap2b | down | 0.24 | 4.72E-08 | transcription factor AP-2 beta |
