# Supplementary material for: Systematic transcriptome analysis of the zebrafish model of diamond-blackfan anemia induced by RPS24 deficiency
Source: BMC Genomics. 2014 Sep 4;15(1):759. doi: 10.1186/1471-2164-15-759 (PMC4169864; doi:10.1186/1471-2164-15-759)
Supplement: Supplementary file 7 — Additional file 7: Table S7: Central nodes of the regulatory network constructed by regulated genes and miRNAs in RPS24 MO. (DOC 31 KB) [file 12864_2014_6455_MOESM7_ESM.doc]

Additional file 7: Table S7 Central nodes of the regulatory network constructed by regulated genes and miRNAs in *RPS24* MO

| Connectivity | Gene Symbol | Description |
| --- | --- | --- |
| 51 | atp6v0a1b | ATPase, H+ transporting, lysosomal V0 subunit a isoform 1b |
| 30 | pank1b | pantothenate kinase 1b |
| 31 | fdps | farnesyl diphosphate synthase (farnesyl pyrophosphate synthetase, dimethylallyltranstransferase, geranyltranstransferase) |
| 37 | h2afv | H2A histone family, member V |
| 34 | zgc:153426 | zgc:153426 |
| 30 | atp6v0cb | ATPase, H+ transporting, lysosomal, V0 subunit c, b |
| 36 | cnot6 | CCR4-NOT transcription complex, subunit 6 |
| 63 | nsfa | N-ethylmaleimide-sensitive factor a |
| 43 | gnb1b | guanine nucleotide binding protein (G protein), beta polypeptide 1b |
