# Supplementary material for: Systematic transcriptome analysis of the zebrafish model of diamond-blackfan anemia induced by RPS24 deficiency
Source: BMC Genomics. 2014 Sep 4;15(1):759. doi: 10.1186/1471-2164-15-759 (PMC4169864; doi:10.1186/1471-2164-15-759)
Supplement: Supplementary file 8 — Additional file 8: Table S8: The primers designed for Real-time PCR. (DOC 32 KB) [file 12864_2014_6455_MOESM8_ESM.doc]

**Additional file 8:** Table S8 The primers designed for Real-time PCR

| **Genes** | **Primers** |
| --- | --- |
| Fos | forward primer: ttacccgctcaaccagactc  reverse primer: agctcgggttgtaggattga |
| Junba | forward primer: ggagagaacttgcggacttt  reverse primer: cgctcatgttctgctttagg |
| Ndrg4 | forward primer: ctccttttcttccctacact  reverse primer: catggtaggtcagaatagct |
| Tbx1 | forward primer: gggatcaagcaggaaacacc  reverse primer: gtcgtgaaggctgcaacatc |
| Hand2 | forward primer: gaccattcgcactacggaggag  reverse primer: aggcgctgttgatgctctgagt |
| Cyp26a1 | forward primer: aggtgaagagcgccatacag  reverse primer: ttccaccagttcttgctcgt |
| β-Actin | forward primer: agatcaagatcattgctccccc  reverse primer: ggccatttaaggtggcaaca |
| Dre-miR-223 | gctgtcagtttgtcaaatacccc |
| Dre-miR-338 | gctccagcatcagtgattttgttg |
| Dre-miR-29a | gctagcaccatttgaaatcggtta |
| Dre-miR-155 | gcttaatgctaatcgtgatagggg |
| Dre-miR-142a-5p | gccataaagtagaaagcactact |
| Dre-miR-736 | gcgtaagacgaacaaaaagtttt |
| Dre-miR-34b | gctaggcagtgttgttagctgattg |
| Dre-miR-2187 | gcgcttaattagtatagcctgtttta |
| Dre-miR-202 | gcttcctatgcatatacctctttg |
| Dre-miR-19b | gcagttttgctggtttgcattcag |
| Dre-miR-142a-3p | gctgtagtgtttcctactttatgga |
